# A Multinodular Mass of Abdominal Splenosis: Case Report of Uncommon Images of a Rare Disease

**DOI:** 10.3390/diagnostics9030111

**Published:** 2019-09-04

**Authors:** Hiroyuki Matsubayashi, Etsuro Bando, Hiroyasu Kagawa, Keiko Sasaki, Hirotoshi Ishiwatari, Hiroyuki Ono

**Affiliations:** 1 Division of Endoscopy, Shizuoka Cancer Center, Suntogun, Shizuoka 411-8777, Japan (H.I.) (H.O.); 2 Division of Gastric Surgery, Shizuoka Cancer Center, Suntogun, Shizuoka 411-8777, Japan; 3 Division of Colon and Rectal Surgery, Shizuoka Cancer Center, Suntogun, Shizuoka 411-8777, Japan; 4 Division of Pathology, Shizuoka Cancer Center, Suntogun, Shizuoka 411-8777, Japan

**Keywords:** abdominal splenosis, splenic injury, diagnosis, multi-nodular mass, EUS-FNA

## Abstract

Splenosis is a rare disease which typically forms single or multiple round masses. A 45-year-old male was referred for investigation of an abdominal mass. He had a history of splenic injury from a traffic accident at age 19. Contrast-enhanced computed tomography showed a well-enhanced, multi-nodular mass lesion, 3.5 cm in size, located below the stomach. An endoscopic ultrasound-guided fine needle aspiration biopsy (EUS-FNAB) for the mass was inconclusive. A surgery was performed, and pathology of the resected mass confirmed splenosis. Clinicians must bear in mind the possibility of occurrence of splenosis after splenic trauma and its image variations.

## 1. Introduction

Splenosis arises from splenic trauma, including splenectomy and physical injury, which results in a portion of the spleen breaking off and embedding itself elsewhere in the body. Consequently, splenosis can develop anywhere the disseminated splenic tissue can be implanted. The splenic masses are often detected incidentally on imaging, but some gastrointestinal splenosis appears with bleeding [1,2,3]. Clinical images of splenosis typically show single or multiple, round or oval masses [4,5,6], which sometimes mimic malignancies such as gastrointestinal stromal tumors (GISTs) [2,3,7], lymphoma [4], peritoneal mesothelioma, renal cancer [5], and hepatocellular carcinoma [8]. In the current case, the patient had a history of a traffic injury and showed a multinodular abdominal splenosis with uncommon images that complicated the diagnosis.

## 2. Case Presentation

A 45-year-old asymptomatic male was referred to our hospital for examination of an abdominal mass that was a suspected mesenteric hemangioma or neuroendocrine tumor (NET). The mass lesion had been incidentally detected by computed tomography done to examine his upper abdominal pain. He had habits of drinking (500 mL of beer per day) and smoking (20 tobacco products per day). He had gallstones and chronic pancreatitis, and showed elevated levels of serum amylase (constantly 150–200 U/L and >500 U/L at attack, normal range: 37–125 U/L). He also had a history of splenic rupture following a traffic accident at the age of 19. His older brother had a history of lung tuberculosis in his 20s. His mother and maternal uncle had died of pancreatic cancer.

**Figure 1:**
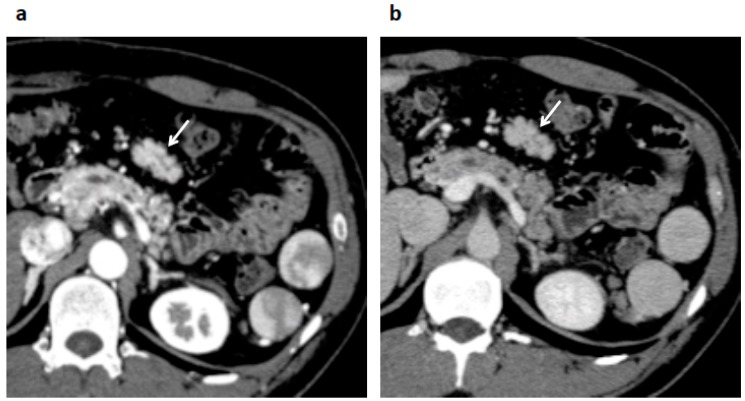
Computed tomography (CT) showing spreading original spleen tissue and an enhanced multinodular lesion (arrow) located anterior to the pancreas body, 35 mm in size, with a vascularity level similar to that of the original spleen at 40 s (**a**) and 70 s (**b**) after contrast injection.

**Figure 2:**
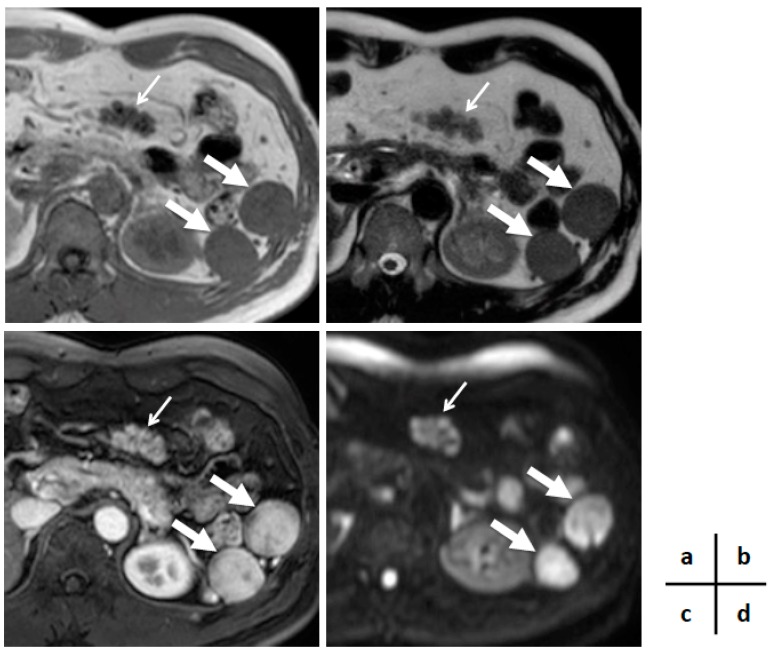
Magnetic resonance imaging (MRI). T1-weighted image (**a**) and T2-weighted image (**b**) demonstrating a low-intensity signal similar to that of the spleen. Enhanced MRI showing a similar enhancement level in the lesion and the spleen (30 s after contrast injection) (**c**). Diffusion-weighted image showing a mass lesion with a heterogeneous diffusion level among multiple nodules (**d**) (thin arrows indicating the lesion and thick arrows indicating the original spleen).

**Figure 3:**
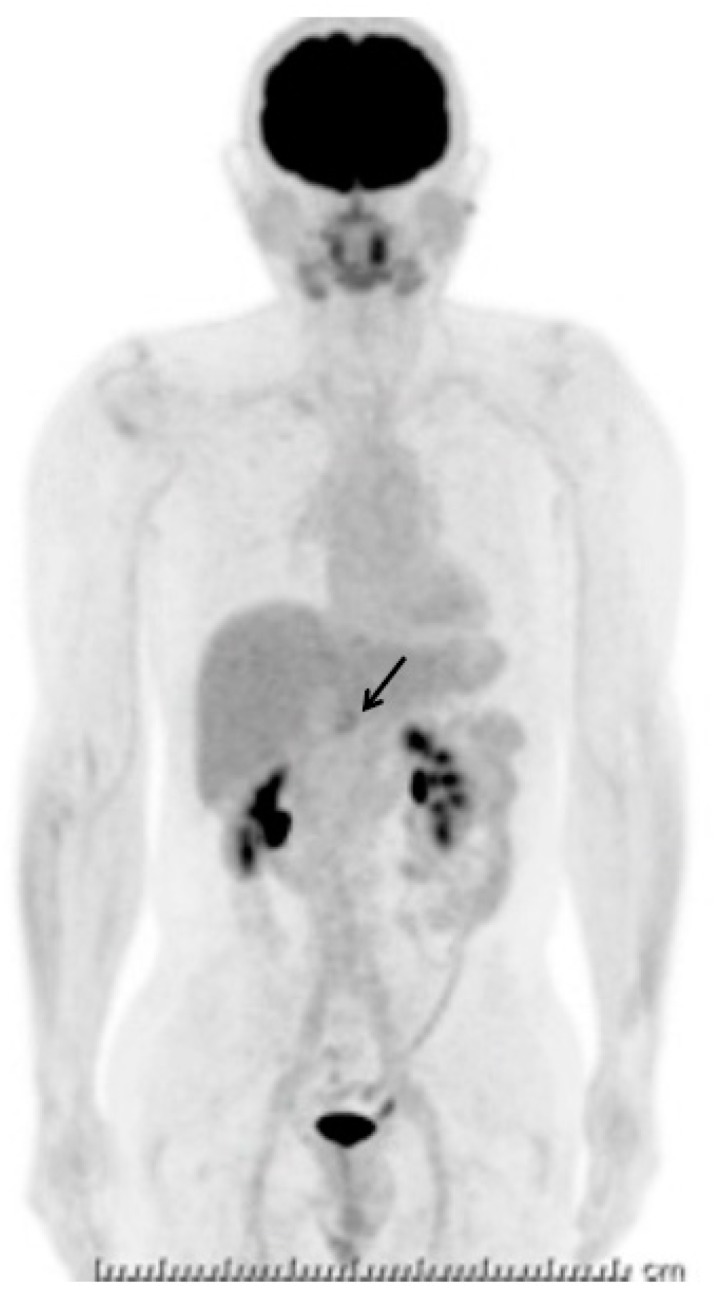
^18^F-fluorodeoxy-glucose positron emission tomography (FDG-PET) showing a faint uptake at the lesion (arrow).

**Figure 4:**
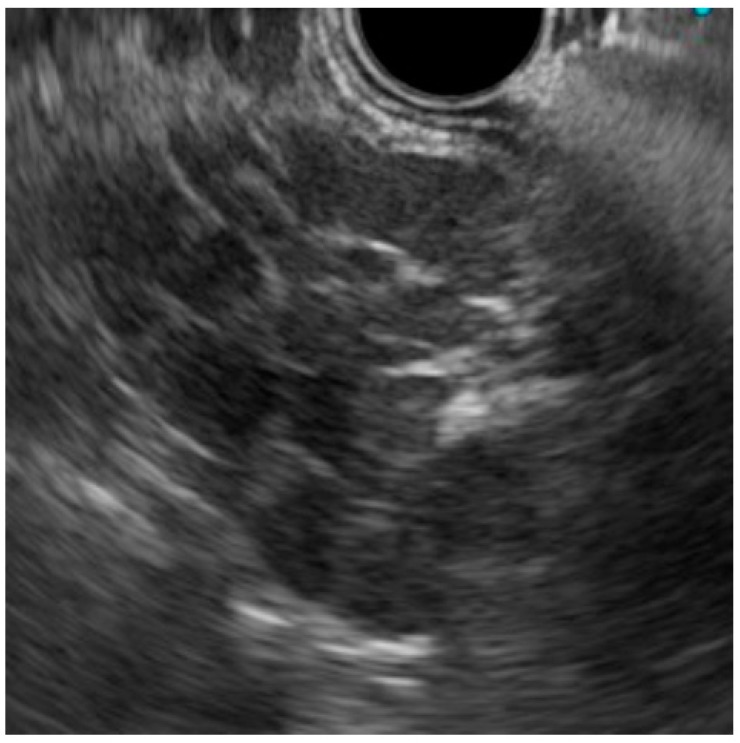
Endoscopic ultrasonography (EUS) demonstrating a mass lesion composed of multiple aggregated low-echoic nodules.

His blood test revealed a slightly elevated serum amylase, but the test was otherwise normal, including readings for several serum tumor markers (carcinoembryonic antigen, cancer antigen 19-9, alpha fetoprotein, neuron-specific enolase, pro-gastrin-releasing peptide, soluble interleukin-2 receptor), and immunoglobulin G and G4. Enhanced computed tomography (CT) demonstrated a well-enhanced multinodular mass lesion, 35 mm in size, located anterior to the pancreas and inferior to the stomach (Figure 1). Magnetic resonance imaging (MRI) (Figure 2) of the mass revealed T1- and T2-weighted image intensities that were almost equal to those of the spleen images. The vascularity of the lesion determined on enhanced-CT and MRI was similar to that of the spleen. Enhancement of the mass and the spleen was measured as 92–158 hansfold units and 95–160 on the CT, at 70th seconds after contrast injection (Figure 1b). However, the intensity of a diffusion-weighted image was heterogeneous within the mass, with high intensity areas corresponding to the nodules. ^18^F-fluorodeoxy-glucose positron emission tomography (FDG-PET) demonstrated a weak uptake at the lesion (Figure 3). Endoscopic ultrasound (EUS) revealed a mass composed of multiple aggregated low-echoic nodules capsulized by a high-echoic septum (Figure 4). These image findings raised a variety of candidate diagnoses, including lymphoma, Castleman’s disease, tuberculous lymphadenitis, splenosis, heterotopic pancreas, solitary fibrous tumor, desmoids, NET, and GIST.

EUS-guided fine needle aspiration (FNA) was performed using a 22-Gauge Franseen needle (Boston Scientific, Marlborough, MA, USA) to make a conclusive diagnosis and a decision on the management strategy. The endosonographer felt a soft touch during the strokes. The obtained tissue revealed aggregated white blood cells without the cellular atypia or monotonous proliferation pattern suggestive of lymphoma. Culture and polymerase chain reaction of the FNA material was negative for acid-fast or tubercle bacilli. The final candidate diagnoses were splenosis, benign lymphoproliferative diseases, lymphadenitis, and indolent-type lymphoma with very weak cytological atypia. These findings and conceivable interpretations were well informed, and the patient chose laparotomy rather than observation.

**Figure 5:**
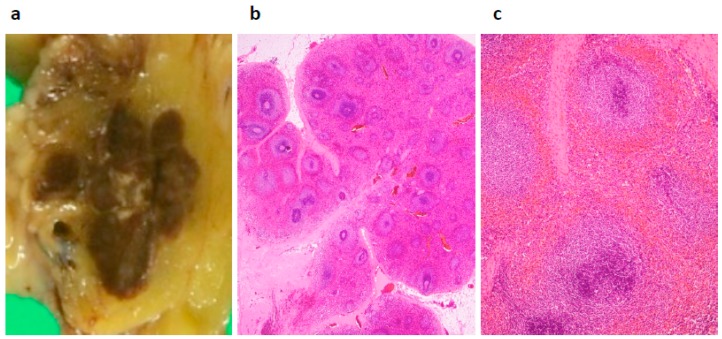
Pathology of the resected material. Tissue slices showing a brownish mass lesion consisting of multiple mm-sized nodules adhering to the colonic mesentery (**a**). Low-power view (**b**) and high-power view (**c**) of the brownish area, showing white pulp, red pulp, and trabecular lienis (Hematoxylin & Eosin staining).

During the laparotomy, the mass lesion was found adhering to the mesentery of transverse colon, so a partial transverse colectomy was performed. Macroscopically, the mass was brownish and well-demarcated consisting of multiple nodules (some mm sized) and was involved in the mesenteric fatty tissue (Figure 5a). Microscopically, the brownish tissue consisted of white pulp, red pulp, and trabecular lienis (Figure 5b,c). Hemosiderin deposition was seen in the red pulp. No malignant cells were seen, and the final diagnosis was splenosis. The post-surgical days were uneventful, and the patient was discharged in a week.

## 3. Discussion

Splenosis is a heterotopic splenic tissue implantation that usually develops after splenic trauma or splenectomy. As such, it is a different entity from congenital malformation or polysplenia [9]. The incidence of splenosis development after splenectomy is <0.3% [8]. The newly embedded ectopic splenic tissue recruits a local blood supply and becomes functional splenic tissue. An accessory spleen can be distinguished from splenosis since an accessory spleen is functionally and histologically similar to normal splenic tissue, whereas splenosis tissue is missing key splenic characteristics, such as a thick capsule, smooth muscle elements, and a blood supply arising from the splenic artery [9]. The ectopically implanted splenic tissue also has a discreetly different architecture, with plenty of red pulp and little white pulp, when compared with the normal spleen [6,9]. For this reason, the image features of the splenosis can differ from those of the original spleen.

In the current case, the intensity of T1- and T2-weighted images and the vascularity of the splenosis were compatible with the spleen, but were not homogeneous. In addition, the vascularity level of this lesion was almost similar to that of spleen (Figure 1 and Figure 2). Hence, careful evaluation of the vascularity of the lesion helps the diagnosis. As the prolonged enhancement is reported to be a key feature of splenosis, contrast-enhanced ultrasonography can be an effective tool to diagnose this disease [10]. Superparamagnetic iron oxide (SPIO) is specifically taken up by reticuloendothelial tissue, such as that found in the spleen. Hence, SPIO MRI is thought as another promising image modality for the diagnosis of this disease [11]. However, the finding of a multinodular mass (Figure 1, Figure 2 and Figure 4) is quite uncommon, as the previously reported findings of splenosis are mostly well-enhanced, round or oval, single [2,12,13,14,15] or multiple [1,3,5,6] mass lesions [3,6,7,9,12,13,14,15]. Only one case reported by Xiao et al. showed a somewhat irregularly margined mass [2]. A common finding in their case and ours was that both cases developed after an abdominal injury, not after splenectomy. No reason for this unique image can be proposed due to the sparsity of similar cases.

The rarity of the images, as indicated above, created difficulties in the preoperative diagnosis of the current case. Several lymphoproliferative lesions and neoplastic lesions were listed as candidates. Due to the patient’s family history, tuberculous lymphadenitis was also suspected and an acid-fast bacillus test was performed on EUS-FNA samples. The EUS-FNA material also excluded several neoplastic diseases and tuberculous infection, although the possibility of Castleman’s disease and lymphoma with very weak cytological atypia remained. A laparotomy was ultimately performed upon full informed consent by the patient. However, some cases of splenosis in the gastrointestinal tract and mesentery cause spontaneous bleeding and need emergent transarterial embolization [1,2,3]. Therefore, in a sense, this surgery was considered to be prophylactic management for a middle-aged individual at risk.

In conclusion, the clinicians must bear in mind the possibility of the development of splenosis in cases with a history of splenic rupture or splenectomy. The image findings of splenosis can vary, so tissue acquisition is worthwhile after evaluation of all the image information.

## Abbreviations

GIST	gastrointestinal stromal tumor
NET	neuroendocrine tumor
CT	computed tomography
MRI	magnetic resonance imaging
FDG-PET	^18^F-fluorodeoxyglucose-positron emission tomography
EUS	endoscopic ultrasonography
FNA	fine needle aspiration
SPIO	super paramagnetic iron oxide
